# The role of intraflagellar transport proteins in ciliary assembly and function

**DOI:** 10.1186/2046-2530-1-S1-O9

**Published:** 2012-11-16

**Authors:** GJ Pazour

**Affiliations:** 1 Program in Molecular Medicine, University of Massachusetts Medical School, USA

## 

The sensory and motility functions of cilia play critical roles in the development of vertebrates and defects in these organelles lead to a wide range of structural birth defects. The intraflagellar transport (IFT) system is required for building all types of mammalian cilia. IFT particles are composed of about 20 proteins and these proteins are highly conserved across ciliated species. IFT25 and IFT27, however, are absent from certain ciliated organisms like *Caenorhabditis* and *Drosophila* suggesting that they may have a unique role distinct from ciliogenesis. We generated *Ift25* and *Ift27* null mice and show that these proteins are not required for ciliary assembly but are required for proper Hedgehog signaling, which in mammals occurs within cilia. Mutant mice die at birth with multiple phenotypes indicative of Hedgehog signaling dysfunction. Cilia lacking IFT25/27 have defects in the signal-dependent transport of multiple Hedgehog components including Patched-1, Smoothened, and Gli2 and fail to activate the pathway upon stimulation. These are the first examples of null IFT mutations that perturb Hedgehog signaling independent of ciliary architecture. Thus, IFT function is not restricted to assembling cilia where signaling occurs but also plays a direct role in signal transduction events.

